# Sepsis-induced coagulopathy (SIC) in the management of sepsis

**DOI:** 10.1186/s13613-024-01380-5

**Published:** 2024-09-20

**Authors:** Toshiaki Iba, Julie Helms, Jerrold H. Levy

**Affiliations:** 1 Department of Emergency and Disaster Medicine, Juntendo University Graduate School of Medicine, 2-1-1 Hongo Bunkyo-ku, Tokyo, 113-8421 Japan; 2 Medical Intensive Care Unit - NHC, Strasbourg University (UNISTRA), Strasbourg University Hospital, INSERM (French National Institute of Health and Medical Research), UMR 1260, Regenerative Nanomedicine (RNM), FMTS, Strasbourg, France; 3 Department of Anesthesiology, Critical Care, and Surgery, Duke University School of Medicine, Durham, NC USA

**Keywords:** Sepsis, Disseminated intravascular coagulation, Coagulopathy, Anticoagulants, Clinical trial

## Abstract

The mortality rate of sepsis remains high and further increases when complicated by disseminated intravascular coagulation (DIC). Consequently, early detection and appropriate management of DIC will be helpful for the management of sepsis. Although overt DIC criteria are often used for diagnosing definitive DIC, it was not designed to detect early-phase DIC. The criteria and scoring system for sepsis-induced coagulopathy (SIC) were developed and introduced in 2017 to detect early-stage DIC, and they were subsequently adopted by the International Society on Thrombosis and Haemostasis in 2019. The objective of detecting SIC was not to miss the patients at high risk of developing overt DIC at an earlier time. Although anticoagulant therapies are potential options for the treatment of sepsis-associated DIC, their effectiveness has not been established, and further research is warranted. For that purpose, an international collaborative platform is required for future clinical trials, and SIC criteria have been suggested for such studies. Calculating the SIC score is straightforward and suitable for use in clinical settings. This review aims to introduce SIC criteria and its scoring system for better management of sepsis-associated DIC. We also intended to update the current knowledge regarding this novel diagnostic criterion.

## Introduction

Sepsis is defined as life-threatening organ dysfunction due to a dysregulated host response to infection [1]. The definition of sepsis was updated in 2016, and the Third International Consensus Definitions for Sepsis and Septic Shock (sepsis-3) has become the current standard [2]. Following this, sepsis-induced coagulopathy (SIC) criteria and its scoring system were constructed in 2017 to categorize coagulopathy in sepsis [3]. Subsequently, the Scientific Standardization Committee on Disseminated Intravascular Coagulopathy (DIC) of the International Society on Thrombosis and Haemostasis (ISTH) adopted SIC for the diagnosis of early-phase DIC in 2019 [4]. After that, the SIC scoring system has been used to screen and diagnose DIC in sepsis globally [5].

**Fig. 1:**
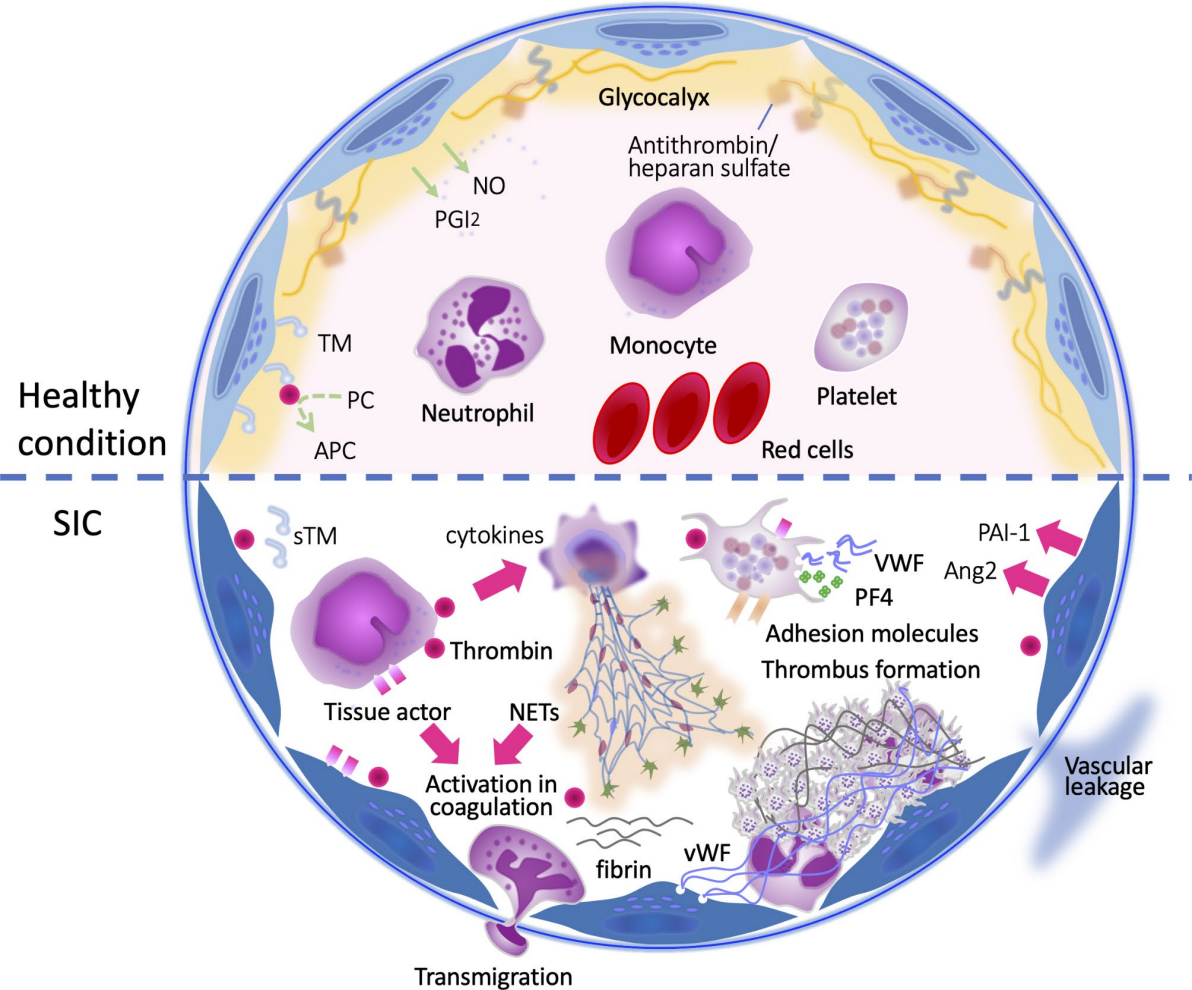
Antithrombogenicity in physiological status and prothrombotic shift in sepsis-induced coagulopathy The vascular endothelial cells maintain the antithrombotic condition by producing nitric oxide (NO) and prostaglandin I_2_ (PGI_2_). Antithrombin/heparan sulfate of the endothelial glycocalyx contributes to the antithrombogenicity of the luminal surface. Thrombomodulin (TM) on the surface of endothelial cells converts protein C (PC) to activated protein C (APC) and exerts antithrombotic activity. This antithrombotic condition turns to the opposite in sepsis-induced coagulopathy (SIC). Monocyte expresses tissue factor (TF) and initiates the extrinsic coagulation cascade, and exposed collagen beneath the endothelium initiates the intrinsic coagulation cascade. Activated neutrophils eject neutrophil extracellular traps (NETs) and further propagate coagulation and inflammation. Platelets also participate in thrombus formation by releasing von Willebrand factor (VWF) and platelet factor 4 (PF4). Damaged endothelium releases VWF, angiopoietin 2 (Ang2), and expresses adhesion molecules that facilitate cellular attachment. Endothelial cells produce excess plasminogen activator inhibitor 1 (PAI-1) and suppress fibrinolysis

Early detection of coagulation disorders is crucial for assessing the severity and predicting the prognosis of sepsis [6]. Recent studies have demonstrated that inflammation and coagulation collaboratively contribute to the pathogenesis of organ dysfunction [7]. Activated leukocytes, platelets, and endothelial damage are also known to play critical roles in thromboinflammation in sepsis [8]. As a consequence, microthrombi formed in the capillaries lead to tissue malcirculation and subsequent organ dysfunction in sepsis [9] (Fig. 1). Despite the advancements mentioned above in understanding the pathophysiology of DIC in sepsis, progress in management has stagnated [10]. Circulatory shock and DIC are the major drivers that deteriorate tissue oxygen supply, and the early diagnosis and resuscitation of shock are essential to improve the outcome of patients with sepsis [11]. Similarly, we think the early management of DIC is critical [3–9]. In this review, we suggest potential strategies to tackle this challenging condition by introducing a novel DIC scoring system.

## Pathophysiology of coagulopathy in sepsis

In response to an infection, sequential events disrupt the homeostasis of blood coagulation, fibrinolysis, and anticoagulation, resulting in detrimental coagulopathy [8]. First, widespread inflammation is triggered in response to infection, and the proinflammatory cytokines, chemokines, and mediators such as tumor necrosis factor-α (TNF-α), interleukin-1β (IL-1β), IL-6, IL-8, and proteolytic enzymes are released. These inflammatory cytokines and mediators promote prothrombotic change in the myeloid leukocytes. An experimental model has shown that intravascular activation of the extrinsic coagulation pathway via tissue factor derived from myeloid leukocytes, such as monocytes and neutrophils, causes fibrin formation. Additionally, thrombus-resident neutrophils are indispensable for thrombus propagation by activating the intrinsic pathway through factor XII activation via the release of neutrophil extracellular traps (NETs) [12]. The above response is not only triggered by the pathogen or pathogen-associated molecular patterns (PAMPs) but also propagated by the host cell-derived damage-associated molecular patterns (DAMPs). Notably, these procoagulant changes are further perturbated by released microvesicles, which express characteristics similar to those of the mother cells from the activated or injured cells [13]. Simultaneously, inflammation and activated coagulation damage the vascular endothelial cells. Inflammation-derived mediators include matrix metalloproteinases, heparanase, hyaluronidase, thrombin, elastase, and reactive oxygen species disrupt endothelial glycocalyx [14], and alter the expression of surface molecules, including tissue factor, adhesion molecules, and von Willebrand factor (VWF). Adhesion molecules such as intercellular adhesion molecule (ICAM), vascular cell adhesion molecule (VCAM), and E-selectin play pivotal roles in monocytes, neutrophils as well as platelets adhesion to the endothelial surface, leading to the intraluminal microthrombi formation. Angiopoietin-2 and plasminogen activator inhibitor-1 (PAI-1) released from endothelial cells also regulate inflammation, coagulation, fibrinolysis, and cell-cell interactions [15]. Angiopoietin-2, a partial antagonist of the endothelium-stabilizing receptor Tie-2, contributes to adverse outcomes in this disease by suppressing vascular endothelial cell survival, reducing vascular endothelial barrier, and increasing vascular permeability and leukocyte migration into tissues [16]. In sepsis, activation and aggregation of platelets are key events. Fibrin formation bursts on the activated platelet surface, and platelet adhesion to neutrophils stimulates NETs release and forms an immunothrombus [17]. Activated platelets adhere to damaged endothelium and release prothrombotic mediators such as p-selectin, soluble C-type lectin-like receptor 2 (CLEC-2), and VWF, further promoting thromboinflammation [18].

Unbalanced fibrinolysis is another characteristic feature of sepsis. Initially, the release of tissue-type plasminogen activator may lead to temporal hyperfibrinolysis. This is ultimately overcome by excess production of PAI-1 and degradation of plasminogen by neutrophil elastase from NETs, leading to insufficient plasmin formation [19], thus contributing to the fibrinolytic suppression and formation of microvascular thrombi [20].

With the changes in coagulation and fibrinolysis, natural anticoagulant pathways, such as the heparan sulfate-antithrombin and thrombomodulin-protein C systems, become impaired. These changes play crucial roles in exacerbating the already imbalanced coagulation/fibrinolytic equilibrium. As was shown, understanding the pathophysiology of coagulopathy in sepsis is critical in developing targeted therapies to modulate the coagulation cascade and improve outcomes in septic patients [21].

Uncontrolled activation of the coagulation finally leads to DIC. The terminal stage DIC is characterized by widespread microvascular thrombosis, consumption of clotting factors and platelets, and ultimately may cause thrombotic events simultaneously with bleeding complications due to depletion of coagulation factors [22].

## Clinical assessment of SIC

### Concept of establishing SIC criteria

Expanding the number of tests and devising a more intricate scoring system for precise diagnosis is typically straightforward. Nonetheless, such a scoring system may not gain widespread use [23, 24]. The primary features of the SIC scoring system are simplicity and ease of calculation. SIC criteria include only two coagulation markers that are readily available in routine, can be rapidly measured, and are easy to calculate at the bedside and in the emergency room. Since both tests are not costly, SIC scoring is also suitable for repetitive measurement and monitoring of the disease’s progress in developing countries [24]. Although the simplest diagnostic criterion, Tsantes et al. [25] argued that SIC had demonstrated adequate sensitivity and specificity in identifying patients at risk for DIC.

On the other hand, the drawback of SIC criteria is its relatively low specificity. Rare but severe conditions, such as patients with cirrhosis, heparin-induced thrombocytopenia, and thrombotic microangiopathy, need to be differentiated in diagnosing SIC [26]. Other than that, it should be noted that SIC is not homogeneous, with characteristics varying based on patient demographics, comorbidities, underlying diseases, and failed organs [27, 28]. Finally, while SIC criteria facilitate timely diagnosis before the decompensated stage of coagulopathy, there is a risk that it may detect numerous cases of mild or non-progressive coagulopathy.

### Constructing a scoring system for SIC

The members of the DIC Scientific Standardization Committee members of the ISTH developed the SIC criteria and the scoring system. For the derivation cohort, a total of 1,498 septic patients with coagulopathy who were treated with recombinant thrombomodulin were utilized. Through univariate and multivariate analyses, variables independently associated with 28-day mortality were identified. As a result, the platelet count, PT, and SOFA score emerged as independent predictors of a fatal outcome. The diagnosis of SIC is based on decreased platelet count: 1 point if 100–150 × 10^9^/L, 2 points if < 100 × 10^9^/L; prothrombin time/international normalized ratio (PT-INR): 1 point if 1.2–1.4, 2 points if > 1.4; and the sequential organ failure assessment (SOFA) score calculated by the sum of respiratory, hepatic, cardiovascular, and renal dysfunction scores: 1 point if 1, 2 points if ≥ 2. The patients are diagnosed as SIC when the total score is 4 or more. Since the total SOFA score was defined as 2 if the total score exceeded 2, the total platelet count and PT-INR must exceed 2 for the diagnosis of SIC [4] (Table 1). Ultimately, platelet count is useful as it is a routine test and effective for screening coagulation disorder, and PT-INR is helpful since it correlates well with the severity of sepsis [29]. The SOFA should be more than two in patients with sepsis-3, which may not be necessary. Nonetheless, it remains valuable to monitor the trajectory of SIC scores by calculating SIC daily during sepsis.

**Table 1:** ISTH overt DIC, JAAM DIC, and SIC scoring systems

		ISTH overt DIC	JAAM DIC	ISTH SIC
Item	Score	Range	Range	Range
Platelet count ( x10^9^/L)	3	−	< 80 or ≧ 50% decrease within 24 h	−
	2	< 50	−	< 100
	1	≧ 50, < 100	120 >, 80 ≦ or ≧ 30% decrease within 24 h	≧ 100, < 150
FDP (D-dimer)	3	strong increase	≧ 25 μg/mL (use convert chart)	−
	2	moderate increase	−	−
	1	−	≧ 10, < 25 μg/mL (use convert chart)	−
Prothrombin time (PT)	2	≧ 6 s	−	> 1.4
	1	≧ 3 s, < 6 s	≧ 1.2 (PT ratio)	> 1.2, ≦ 1.4 (PT-INR)
Fibrinogen (g/mL)	1	< 100	−	−
SIRS score	1	−	> 3	−
SOFA score	2	−	−	≧ 2
	1	−	−	1
Total score for DIC or SIC		≧ 5	≧ 4	≧ 4

ISTH: International Society on Thrombosis and Haemostasis; DIC: disseminated intravascular coagulation; JAAM: Japanese Society for Acute Medicine; SIC: Sepsis-induced coagulopathy; SIRS: Systemic Inflammatory Response Syndrome; SOFA: sequential organ failure assessment; INR: international normalized ratio

Total SOFA score is the sum of 4 items (respiratory SOFA, cardiovascular SOFA, hepatic SOFA, and renal SOFA). The score of total SOFA was defined as 2 if the total score exceeded 2, and the total score of platelet count and PT-INR must exceed 2 for the diagnosis of SIC

### Prevalence and mortality of SIC

The secondary analysis of two randomized controlled trials examined the prevalence and mortality of patients with SIC- according to the sepsis-3 definition [30]. According to the report, the prevalence of SIC was 22.1% (95% confidence interval [CI], 17.5–27.5%) in the Effect of Hydrocortisone on Development of Shock Among Patients With Severe Sepsis (HYPRESS) trial, and 24.2% (95% CI, 21.6–26.9%) in the Effect of Sodium Selenite Administration and Procalcitonin-Guided Therapy on Mortality in Patients With Severe Sepsis or Septic Shock (SISPCT) trial. The 90-day mortality of patients with sepsis-3 and SIC without shock was significantly higher and almost doubled compared to that in the patients without (26.8% vs. 13.9%, *p* = 0.027) in the HYPRESS trial. Most importantly, the presence of SIC was early and presented at sepsis diagnosis or occurred in the following 4 days.

While DIC is characterized by systemic coagulation activation leading to endothelial damage [31], SIC criteria do not encompass endothelial markers. Therefore, incorporating endothelial damage indicators like VWF and antithrombin activity can present an intriguing approach to enhancing the performance of SIC [32, 33]. Li et al. [34] also reported the performance of SIC could be improved by combining it with endothelial cell-related molecular markers, such as soluble thrombomodulin, PAI-1, and angiopoietin-2.

### SIC in practice

How commonly are SIC criteria used? Since the release of SIC, the Scientific and Standardization Committee of the ISTH has continuously supported using SIC criteria and proposed a two-step approach using SIC and overt DIC criteria [5]. In addition, the European Society of Cardiology Working Group and ISTH announced a Joint clinical consensus statement on ongoing antithrombotic therapy for hospitalized patients with severe infection [35]. This statement focuses on the application of combined therapy with antiplatelets and/or anticoagulants in severe infections of bacterial and viral etiology and refers to the SIC criteria for the diagnosis of coagulopathy. This consensus statement also indicated the SIC score-guided antithrombotic therapy. Besides, the Japanese Clinical Practice Guidelines for Management of Sepsis and Septic Shock were updated, and version 2024 (https://www.jsicm.org/news/news210225.html) introduce SIC criteria together with the Japanese Association for Acute Medicine (JAAM) DIC criteria and overt DIC criteria.

The mortality of septic patients increases with the development of SIC, and we think calculating the SIC score helps ICU physicians recognize the severity of the patients. SIC diagnosis is also useful for identifying patients at high risk of developing overt DIC at an early timing. Although direct evidence that showed SIC diagnosis improved outcomes is still lacking, Umemura et al. [36] analyzed data from 2,663 patients with severe sepsis and reported that DIC screening was associated with a reduction in mortality. This is likely because diagnosing DIC prompted physicians to prepare for difficult cases.

Since sepsis is a heterogeneous group of patients with infection, a uniform approach will not be appropriate. Precision medicine principles should be applied to select suitable candidates [37]. SIC may be a potential tool for selecting patients with sepsis who are suitable for anticoagulation therapy.

### Comparison to other criteria

**Fig. 2:**
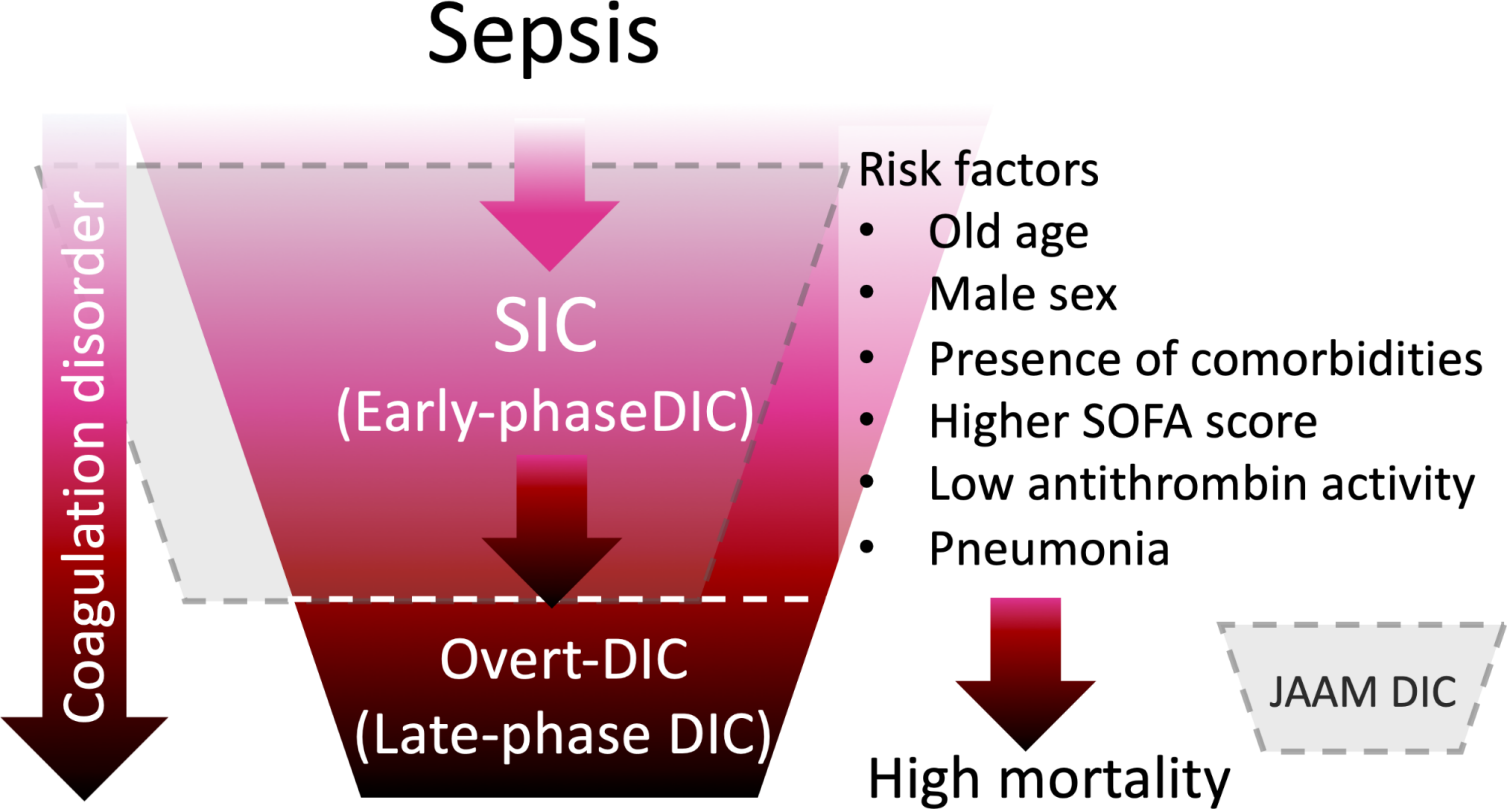
Progression from sepsis to disseminated intravascular coagulation Inflammation and coagulation are the major drivers of disease progression in sepsis. Patients progress from sepsis-induced coagulopathy (SIC), an early-phase disseminated intravascular coagulation (DIC), to overt, late-phase DIC. Multiple risk factors are known to facilitate disease progression and increase the risk of death. The Japanese Association for Acute Medicine (JAAM) DIC criteria is also designed to diagnose early-phase DIC; however, it does not overlap with SIC

How do SIC criteria differ from other commonly used criteria for early-phase DIC? The concept of SIC diagnosis was to identify early-phase DIC that progresses to overt DIC using readily available markers with the simplest approach (Fig. 2). The most popularly used diagnostic criteria for early-phase DIC were released from the JAAM, which was independently developed from the ISTH overt DIC criteria. Consequently, overt DIC may not necessarily represent the continuum of JAAM DIC. The JAAM DIC scoring system comprises platelet count, prothrombin time ratio, fibrin/fibrinogen degradation products, and systemic inflammatory response syndrome (SIRS) score [37]. However, the prognostic accuracy of D-dimer is not high enough because of the suppressed fibrinolysis [38]. Chen et al. [39] examined the relationship between SIC and JAAM DIC scoring systems and 28-day mortality in 452 cases. As a result, a significant difference was seen in the positive rate of SIC between the survivors and the non-survivors (20.0% vs. 38.6%, *p* < 0.001), while the difference was not significant in the JAAM DIC score (42.8% vs. 49.2%, *p* = 0.211). In addition, Li et al. [40] reported a comparable predictive accuracy for 28-day mortality of the SIC score to the SOFA score in a prospective study. Lyons et al. [41] proposed sepsis-associated coagulopathy (SAC) criteria composed of platelet count and PT-INR under a concept similar to SIC. Zhao et al. [36, 42] retrospectively compared the performance of SAC and SIC in 419 patients with sepsis and reported the specificity of SIC for identifying overt DIC was significantly higher than that of the SAC criteria from day 1 to day 14 (*p* < 0.05).

### Utility in determining treatment

While achieving accurate predictive performance is desirable, the primary aim of the diagnostic criteria is not to differentiate between survivors and non-survivors. The most important clinical query that needs to be answered is, “Is DIC diagnosis appropriate for initiating anticoagulant therapy?” [23, 24]. Czempik et al. [43] discussed the importance of identifying SIC, as anticoagulants may offer the greatest benefit during this early stage of DIC. However, it is challenging to provide a definitive answer for the appropriateness of intervention timing because the effectiveness of anticoagulation in sepsis-associated DIC has yet to be confirmed [44, 45]. In the meta-analyses, improved survival was reported using antithrombin and thrombomodulin for sepsis-associated DIC [46, 47]. However, more robust evidence is needed [48]. The use of anticoagulants for DIC is considerably different between the countries, the Japanese Clinical Practice Guidelines for Management of Sepsis and Septic Shock recommend the use of antithrombin or recombinant thrombomodulin [49], while they were not recommended in the rest of the world [11].

**Fig. 3:**
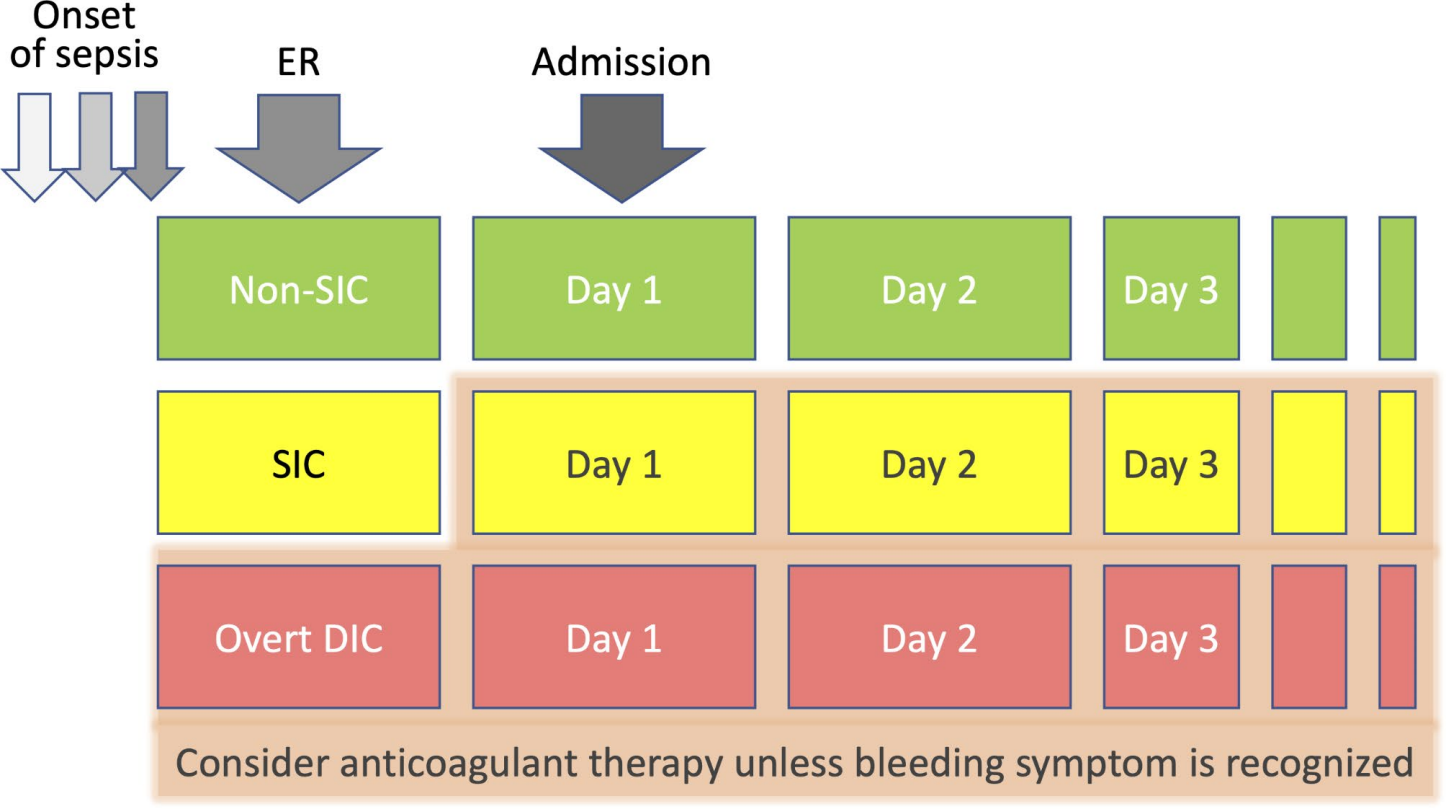
Time course of coagulopathy and application of the anticoagulant therapy The presence or absence of sepsis-induced coagulopathy (SIC) is assessed in the emergency room (ER). In cases where septic patients are complicated by SIC, the test should be repeated daily. If SIC persists or coagulopathy worsens to overt disseminated intravascular coagulation (DIC), integrated management, including anticoagulant therapy, should be considered

For the treatment for SIC, Yamakawa et al. [50] reported better survival with the anticoagulants, i.e., antithrombin and recombinant thrombomodulin, in patients with SIC, but such an effect was not observed in patients without SIC. Although the appropriateness of SIC in detecting patients for anticoagulant therapy needs to be confirmed in prospective randomized trials, we think this type of new challenge will overcome the obstacles of the current sepsis study. (Fig. 3)

## How does SIC change sepsis management?

Before introducing SIC criteria, DIC was diagnosed by overt-DIC criteria, which delayed diagnosis. As a reminder, the ISTH released non-overt DIC criteria together with overt DIC criteria, and the former criteria were designed to diagnose the early-phase DIC [32]. However, since molecular markers such as TAT, antithrombin, and protein C activities were included in the criteria, non-overt DIC criteria were not practical. As a result, only overt DIC criteria have been used, and DIC is now recognized as an advanced phase of the coagulation disorder. Nevertheless, DIC does not define decompensated hemostatic impairment but rather a systemic activation in coagulation [3]2. Since thromboinflammation is deeply involved in the progression of sepsis, timely detection is important, and SIC diagnosis should be made in the emergency room [51]. Conversely, due to the high prevalence of SIC among patients treated in the ICU and the presence of coagulation disorder prior to ICU admission, the clinical benefits of SIC may be limited [52, 53]. Ultimately, the diagnosis with overt DIC definition cannot help improve the outcome of acute infections, and DIC needs to be detected early (Table 2).

**Table 2:** The clinical benefit of the SIC scoring system

Early identification of DIC [4, 32].	Recognizing sepsis-induced coagulopathy (SIC) allows for the early identification of patients at higher risk of developing coagulation abnormalities and related complications in sepsis, such as disseminated intravascular coagulation (DIC) and organ dysfunction.				
Tailored treatment strategies [43, 50]	SIC assessment enables clinicians to tailor treatment strategies based on individual patient profiles. This may involve initiating anticoagulant therapy or other interventions aimed at preventing further coagulation abnormalities and improving outcomes.				
Monitoring disease progression [40, 57]	Regular monitoring of SIC helps clinicians track the progression of coagulopathy and assess the effectiveness of treatment interventions over time. This allows for timely adjustments to the management plan as needed.				
Risk stratification [5, 30, 55]	SIC evaluation aids in risk stratification, allowing clinicians to identify patients who may require more intensive monitoring or interventions to prevent adverse outcomes related to coagulation abnormalities.				
Enhanced prognostication [30, 64]	Incorporating SIC assessment into sepsis management facilitates more accurate prognostication by considering the impact of coagulopathy on patient outcomes.				

## SIC in clinical trials

Apart from its use in practice, due to its simplicity and ease of scoring, SIC criteria are popularly used to extract cases in retrospective studies [27, 30, 40, 52, 54]. SIC would also be well-suited for assessing eligibility in prospective clinical trials [55, 56]. Importantly, SIC diagnosis detects nearly all cases progressing to overt DIC beforehand, ensuring comprehensive screening of patients and minimizing the risk of overlooking suitable candidates. We have examined the relationship between SIC and overt DIC in 332 septic patients suspected to have DIC. As a result, almost all (149 of 151 patients, 98.7%) overt DIC were diagnosed as having SIC at baseline. In addition, of the 49 patients who developed overt DIC between days 2 and 4, 46 (93.9%) had received a prior diagnosis of SIC. The sensitivity of SIC at baseline for predicting death was higher than that of overt DIC (86.8% vs. 64.5%, *p* < 0.001). Similarly, the sensitivity of SIC to death on days 2, 4, and 7 was significantly higher than those of overt DIC [57]. Another study has also reported the SIC diagnosis was an independent predictor for the development of overt DIC (Odd Ratio [OR], 10.39, (95% CI, 4.08–26.46). Furthermore, SIC at emergency department admission was a predictor of in-hospital mortality, with an OR of 2.28 (95% CI, 1.16–4.48) [51].

SCARLET was the first phase 3 trial that examined the effect of anticoagulants in septic patients with coagulopathy. All the phase 3 trials that examined antithrombin, activated protein C, and recombinant tissue factor pathway inhibitor enrolled the patients with severe sepsis or septic shock [58–60]. SCARLET enrolled 800 septic patients with coagulopathy (platelet count in the range of 30 to 150 × 10^9^/L and PT-INR > 1.4) and concomitant cardiovascular and/or respiratory failure. The result showed no difference in 28-day mortality [61]. Interestingly, thrombomodulin showed more enhanced mortality reduction in the subgroup of patients still with coagulopathy at the time of treatment. Another *post hoc* analysis of SCARLET was performed with patients treated only in France, the country with the largest enrollment, with 19% of the full set analysis [62]. 28-day mortality was lower in France, probably because the patients who had a coagulopathy at the time they received treatment were larger. Therefore, screening and monitoring the coagulation status tightly during trials would be vital .

Given the variability in the effectiveness of different anticoagulants [46, 47, 63], determining the optimal target, treatment timing, dose, and duration may vary depending on the specific agent used [64]. We expect employing a repeated measurement approach, such as SIC, could be suitable for establishing personalized optimal treatment strategies [65] (Table 3).

**Table 3:** The benefit of the SIC scoring system in clinical trials

Standardized assessment [4, 5]	SIC criteria provide a standardized framework for assessing coagulation abnormalities in patients with sepsis. This consistency in evaluation allows for more reliable comparisons across different study populations and interventions.
Patient stratification [49]	SIC criteria enable researchers to stratify patients based on their coagulation status, which can help identify subgroups of patients who may benefit more from specific interventions. This stratification enhances the precision of clinical trial results and facilitates personalized medicine approaches.
Outcome prediction [30, 39]	Incorporating SIC criteria into clinical trials allows for the evaluation of their predictive value regarding patient outcomes, such as mortality, organ dysfunction, or thrombotic events. This information can help refine risk stratification models and inform clinical decision-making.
Evaluation of treatment effects [57]	By assessing changes in SIC status over the course of a clinical trial, researchers can evaluate the impact of interventions on coagulation abnormalities. This analysis provides insights into the mechanisms of action of the interventions and their effects on patient physiology.
Endpoint selection [61]	SIC criteria can serve as endpoints or surrogate markers in clinical trials evaluating treatments targeting coagulopathy in sepsis. Using SIC-related endpoints allows for more clinically relevant assessments of treatment efficacy and safety.

## Conclusion

SIC is simple, easy to calculate, and suitable for diagnosis in emergency settings and repeated measurements in the ICU. About 25% of septic patients experience complications with SIC from the early stage, and approximately 25% of SIC patients do not survive. Consequently, as Schmoch et al. [30] described, the occurrence of SIC is associated with higher morbidity and mortality and should be interpreted as an early warning sign. Additionally, monitoring SIC over time can provide valuable information on the progression of the condition and will be suitable for screening candidates for clinical trials. Anticoagulant therapy for patients with SIC is an important consideration; however, its efficacy should be examined in future trials.

## Data Availability

Not applicable.

## Abbreviations

CLEC-2: C-type lectin-like receptor 2
DAMP: Damage-associated molecular patterns
DIC: Disseminated intravascular coagulation
IL: Interleukin
INR: International normalized ratio
ISTH: International Society on Thrombosis and Haemostasis
JAAM: Japanese Association for Acute Medicine
LMWH: Low-molecular-weight heparin
NET: Neutrophil extracellular traps
PAI-1: Plasminogen activator inhibitor-1
PAMP: Pathogen-associated molecular pattern
PT: Prothrombin time
SIC: Sepsis-induced coagulopathy
SIRS: Systemic inflammatory response syndrome
SOFA: Sequential organ failure assessment
TNF: Tumor necrosis factor
VWF: Von Willebrand factor

## References

[1] Rudd KE, Johnson SC, Agesa KM, Shackelford KA, Tsoi D, Kievlan DR, Colombara DV, Ikuta KS, Kissoon N, Finfer S, Fleischmann-Struzek C, Machado FR, Reinhart KK, Rowan K, Seymour CW, Watson RS, West TE, Marinho F, Hay SI, Lozano R, Lopez AD, Angus DC, Murray CJL, Naghavi M. Global, regional, and national sepsis incidence and mortality, 1990–2017: analysis for the global burden of disease study. Lancet. 2020;395(10219):200–11. 31954465 10.1016/S0140-6736(19)32989-7 PMC6970225

[2] Singer M, Deutschman CS, Seymour CW, Shankar-Hari M, Annane D, Bauer M. The third international consensus definitions for sepsis and septic shock (sepsis-3). JAMA. 2016;315(8):801–10. 26903338 10.1001/jama.2016.0287 PMC4968574

[3] Iba T, Nisio MD, Levy JH, Kitamura N, Thachil J. New criteria for sepsis-induced coagulopathy (SIC) following the revised sepsis definition: a retrospective analysis of a nationwide survey. BMJ Open. 2017;7(9):e017046. 28963294 10.1136/bmjopen-2017-017046 PMC5623518

[4] Iba T, Levy JH, Yamakawa K, Thachil J, Warkentin TE, Levi M, Scientific and Standardization Committee on DIC of the International Society on Thrombosis and Haemostasis. Proposal of a two-step process for the diagnosis of sepsis-induced disseminated intravascular coagulation. J Thromb Haemost. 2019;17(8):1265–8. 31099127 10.1111/jth.14482

[5] Iba T, Levi M, Thachil J, Helms J, Scarlatescu E, Levy JH. Communication from the scientific and standardization committee of the international society on thrombosis and haemostasis on sepsis-induced coagulopathy in the management of sepsis. J Thromb Haemost. 2023;21(1):145–53. 36695377 10.1016/j.jtha.2022.10.022

[6] Matsuoka T, Yamakawa K, Iba T, Homma K, Sasaki J. Persistent and late-onset disseminated intravascular coagulation are closely related to poor prognosis in patients with sepsis. Thromb Haemost. 2024;124(5):399–407. 37871648 10.1055/a-2196-3630

[7] Helms J, Iba T, Connors JM, Gando S, Levi M, Meziani F, Levy JH. How to manage coagulopathies in critically ill patients. Intensive Care Med. 2023;49(3):273–90. 36808215 10.1007/s00134-023-06980-6

[8] Iba T, Helms J, Levi M, Levy JH. Thromboinflammation in acute injury: infections, heatstroke, and trauma. J Thromb Haemost. 2024;22(1):7–22. 37541590 10.1016/j.jtha.2023.07.020

[9] Iba T, Helms J, Maier CL, Levi M, Scarlatescu E, Levy JH. The role of thromboinflammation in acute kidney injury among patients with septic coagulopathy. J Thromb Haemost. 2024;22(6):1530–40. 38382739 10.1016/j.jtha.2024.02.006

[10] Unar A, Bertolino L, Patauner F, Gallo R, Durante-Mangoni E. Decoding sepsis-induced disseminated intravascular coagulation: a comprehensive review of existing and emerging therapies. J Clin Med. 2023;12(19):6128. 37834771 10.3390/jcm12196128 PMC10573475

[11] Evans L, Rhodes A, Alhazzani W, Antonelli M, Coopersmith CM, French C, Machado FR, Mcintyre L, Ostermann M, Prescott HC, Schorr C, Simpson S, Wiersinga WJ, Alshamsi F, Angus DC, Arabi Y, Azevedo L, Beale R, Beilman G, Belley-Cote E, Burry L, Cecconi M, Centofanti J, Coz Yataco A, De Waele J, Dellinger RP, Doi K, Du B, Estenssoro E, Ferrer R, Gomersall C, Hodgson C, Møller MH, Iwashyna T, Jacob S, Kleinpell R, Klompas M, Koh Y, Kumar A, Kwizera A, Lobo S, Masur H, McGloughlin S, Mehta S, Mehta Y, Mer M, Nunnally M, Oczkowski S, Osborn T, Papathanassoglou E, Perner A, Puskarich M, Roberts J, Schweickert W, Seckel M, Sevransky J, Sprung CL, Welte T, Zimmerman J, Levy M. Surviving sepsis campaign: international guidelines for management of sepsis and septic shock 2021. Intensive Care Med. 2021;47(11):1181–247. 34599691 10.1007/s00134-021-06506-y PMC8486643

[12] von Brühl ML, Stark K, Steinhart A, Chandraratne S, Konrad I, Lorenz M, Khandoga A, Tirniceriu A, Coletti R, Köllnberger M, Byrne RA, Laitinen I, Walch A, Brill A, Pfeiler S, Manukyan D, Braun S, Lange P, Riegger J, Ware J, Eckart A, Haidari S, Rudelius M, Schulz C, Echtler K, Brinkmann V, Schwaiger M, Preissner KT, Wagner DD, Mackman N, Engelmann B, Massberg S. Monocytes, neutrophils, and platelets cooperate to initiate and propagate venous thrombosis in mice in vivo. J Exp Med. 2012;209(4):819–35. 22451716 10.1084/jem.20112322 PMC3328366

[13] Iba T, Ogura H. Role of extracellular vesicles in the development of sepsis-induced coagulopathy. J Intensive Care. 2018;6:68. 30377532 10.1186/s40560-018-0340-6 PMC6194680

[14] Iba T, Maier CL, Helms J, Ferrer R, Thachil J, Levy JH. Managing sepsis and septic shock in an endothelial glycocalyx-friendly way: from the viewpoint of surviving sepsis campaign guidelines. Ann Intensive Care. 2024;14(1):64. 38658435 10.1186/s13613-024-01301-6 PMC11043313

[15] Whitney JE, Zhang B, Koterba N, Chen F, Bush J, Graham K, Lacey SF, Melenhorst JJ, Teachey DT, Mensinger JL, Yehya N, Weiss SL. Systemic endothelial activation is associated with early acute respiratory distress syndrome in children with extrapulmonary sepsis. Crit Care Med. 2020;48(3):344–52. 32058372 10.1097/CCM.0000000000004091 PMC8749338

[16] David S, Mukherjee A, Ghosh CC, Yano M, Khankin EV, Wenger JB, Karumanchi SA, Shapiro NI, Parikh SM. Angiopoietin-2 may contribute to multiple organ dysfunction and death in sepsis. Crit Care Med. 2012;40(11):3034–41. 22890252 10.1097/CCM.0b013e31825fdc31 PMC3705559

[17] McDonald B, Davis RP, Kim SJ, Tse M, Esmon CT, Kolaczkowska E, Jenne CN. Platelets and neutrophil extracellular traps collaborate to promote intravascular coagulation during sepsis in mice. Blood. 2017;129(10):1357–67. 28073784 10.1182/blood-2016-09-741298 PMC5345735

[18] Ishikura H, Irie Y, Kawamura M, Hoshino K, Nakamura Y, Mizunuma M, Maruyama J, Nakashio M, Suzuki-Inoue K, Kitamura T. Early recognition of sepsis-induced coagulopathy using the C2PAC index: a ratio of soluble type C lectin-like receptor 2 (sCLEC-2) level and platelet count. Platelets. 2022;33(6):935–44. 35073814 10.1080/09537104.2021.2019694

[19] Cruz DBD, Helms J, Aquino LR, Stiel L, Cougourdan L, Broussard C, Chafey P, Riès-Kautt M, Meziani F, Toti F, Gaussem P, Anglés-Cano E. DNA-bound elastase of neutrophil extracellular traps degrades plasminogen, reduces plasmin formation, and Tdecreases fibrinolysis: proof of concept in septic shock plasma. FASEB J. 2019;33(12):14270–80. 31682515 10.1096/fj.201901363RRR

[20] Skibsted S, Jones AE, Puskarich MA, Arnold R, Sherwin R, Trzeciak S, Schuetz P, Aird WC, Shapiro NI. Biomarkers of endothelial cell activation in early sepsis. Shock. 2013;39(5):427–32. 23524845 10.1097/SHK.0b013e3182903f0d PMC3670087

[21] Choi Q, Hong KH, Kim JE, Kim HK. Changes in plasma levels of natural anticoagulants in disseminated intravascular coagulation: high prognostic value of antithrombin and protein C in patients with underlying sepsis or severe infection. Ann Lab Med. 2014;34(2):85–91. 24624342 10.3343/alm.2014.34.2.85 PMC3948838

[22] Larsen JB, Aggerbeck MA, Granfeldt A, Schmidt M, Hvas AM, Adelborg K. Disseminated intravascular coagulation diagnosis: positive predictive value of the ISTH score in a Danish population. Res Pract Thromb Haemost. 2021;5(8):e12636. 34938938 10.1002/rth2.12636 PMC8660681

[23] Thachil J, Iba T. Designing the diagnostic criteria for disseminated intravascular coagulation (DIC). Juntendo Med J. 2023;69(6):1–3. 10.14789/jmj.JMJ23-0038-P PMC11153069 38855069

[24] Iba T, Helms J, Connors JM, Levy JH. The pathophysiology, diagnosis, and management of sepsis-associated disseminated intravascular coagulation. J Intensive Care. 2023;11(1):24. 37221630 10.1186/s40560-023-00672-5 PMC10202753

[25] Tsantes AG, Parastatidou S, Tsantes EA, Bonova E, Tsante KA, Mantzios PG, Vaiopoulos AG, Tsalas S, Konstantinidi A, Houhoula D, Iacovidou N, Piovani D, Nikolopoulos GK, Sokou R. Sepsis-induced coagulopathy: an update on pathophysiology, biomarkers, and current guidelines. Life. 2023;13(2):350. 36836706 10.3390/life13020350 PMC9961497

[26] Iba T, Levy JH, Wada H, Thachil J, Warkentin TE, Levi M. Subcommittee on disseminated intravascular coagulation. Differential diagnoses for sepsis-induced disseminated intravascular coagulation: communication from the SSC of the ISTH. J Thromb Haemost. 2019;17(2):415–9. 30618150 10.1111/jth.14354

[27] Liufu R, Chen Y, Wan XX, Liu RT, Jiang W, Wang C, Peng JM, Weng L, Du B. Sepsis-induced coagulopathy: the different prognosis in severe pneumonia and bacteremia infection patients. Clin Appl Thromb Hemost. 2023;29:10760296231219249. 38126337 10.1177/10760296231219249 PMC10748526

[28] Tanaka C, Tagami T, Kudo S, Takehara A, Fukuda R, Nakayama F, Kaneko J, Ishiki Y, Sato S, Kuno M, Unemoto K. Validation of sepsis-induced coagulopathy score in critically ill patients with septic shock: post hoc analysis of a nationwide multicenter observational study in Japan. Int J Hematol. 2021;114(2):164–71. 33895968 10.1007/s12185-021-03152-4 PMC8067778

[29] Wada T, Yamakawa K, Kabata D, Abe T, Fujishima S, Kushimoto S, Mayumi T, Ogura H, Saitoh D, Shiraishi A, Otomo Y, Gando S, JAAM FORECAST Group. Sepsis-related coagulopathy treatment based on the disseminated intravascular coagulation diagnostic criteria: a post-hoc analysis of a prospective multicenter observational study. J Intensive Care. 2023;11(1):8. 36872342 10.1186/s40560-023-00656-5 PMC9985865

[30] Schmoch T, Möhnle P, Weigand MA, Briegel J, Bauer M, Bloos F, Meybohm P, Keh D, Löffler M, Elke G, Brenner T, Bogatsch H, SepNet–Critical Care Trials Group. The prevalence of sepsis-induced coagulopathy in patients with sepsis - a secondary analysis of two German multicenter randomized controlled trials. Ann Intensive Care. 2023;13(1):3. 36635426 10.1186/s13613-022-01093-7 PMC9837358

[31] Taylor FB Jr, Toh CH, Hoots WK, Wada H, Levi M, Scientific Subcommittee on Disseminated Intravascular Coagulation (DIC) of the International Society on Thrombosis and Haemostasis (ISTH). Towards definition, clinical and laboratory criteria, and a scoring system for disseminated intravascular coagulation. Thromb Haemost. 2001;86(5):1327–30. 11816725

[32] Iba T, Levy JH, Thachil J, Susen S, Levi M, Scarlatescu E. Communication from the scientific standardization committees of the international society on thrombosis and haemostasis on vascular endothelium-related biomarkers in disseminated intravascular coagulation. J Thromb Haemost. 2023;21(3):691–9. 36696178 10.1016/j.jtha.2022.11.032

[33] Helms J, Iba T, Angles-Cano E. Harnessing the power of hemostasis testing in intensive care unit. Intensive Care Med. 2024;50(7):1146–8. 38695927 10.1007/s00134-024-07430-7

[34] Li Y, Li H, Wang Y, Guo J, Zhang D. Potential biomarkers for early diagnosis, evaluation, and prognosis of sepsis-induced coagulopathy. Clin Appl Thromb Hemost. 2023;29:10760296231195089. 37605466 10.1177/10760296231195089 PMC10467369

[35] Gigante B, Levy JH, van Gorp E, Bartoloni A, Bochaton-Piallat ML, Bäck M, Ten Cate H, Christersson C, Ferreiro JL, Geisler T, Lutgens E, Schulman S, Storey RF, Thachil J, Vilahur G, Liaw PC, Rocca B. Management of patients on antithrombotic therapy with severe infections: a joint clinical consensus statement of the ESC working group on thrombosis, the ESC working group on atherosclerosis and vascular biology, and the international society on thrombosis and haemostasis. Eur Heart J. 2023;44(32):3040–58. 37439553 10.1093/eurheartj/ehad388

[36] Umemura Y, Yamakawa K, Hayakawa M, Hamasaki T, Fujimi S. Japan septic disseminated intravascular coagulation (J-Septic DIC) study group. Screening itself for disseminated intravascular coagulation may reduce mortality in sepsis: a nationwide multicenter registry in Japan. Thromb Res. 2018;161:60–6. 29202320 10.1016/j.thromres.2017.11.023

[37] Gando S, Iba T, Eguchi Y, Ohtomo Y, Okamoto K, Koseki K, Mayumi T, Murata A, Ikeda T, Ishikura H, Ueyama M, Ogura H, Kushimoto S, Saitoh D, Endo S, Shimazaki S. Japanese association for acute medicine disseminated intravascular coagulation (JAAM DIC) study group. A multicenter, prospective validation of disseminated intravascular coagulation diagnostic criteria for critically ill patients: comparing current criteria. Crit Care Med. 2006;34(3):625–31. 16521260 10.1097/01.ccm.0000202209.42491.38

[38] Schupp T, Weidner K, Rusnak J, Jawhar S, Forner J, Dulatahu F, Brück LM, Hoffmann U, Kittel M, Bertsch T, Akin I, Behnes M. D-dimer levels and the disseminated intravascular coagulation score to predict severity and outcomes in sepsis or septic shock. Clin Lab. 2023;69(5). 10.7754/Clin.Lab.2022.221015 37145079

[39] Chen Y, Chen W, Ba F, Zheng Y, Zhou Y, Shi W, Li J, Yang Z, Mao E, Chen E, Chen Y. Prognostic accuracy of the different scoring systems for assessing coagulopathy in sepsis: a retrospective study. Clin Appl Thromb Hemost. 2023;29:10760296231207630. 37920943 10.1177/10760296231207630 PMC10623916

[40] Li J, Liu H, Wang N, Wang F, Shang N, Guo S, Wang G. Persistent high sepsis-induced coagulopathy and sequential organ failure assessment scores can predict the 28-day mortality of patients with sepsis: a prospective study. BMC Infect Dis. 2024;24(1):282. 38438863 10.1186/s12879-024-09154-x PMC10913246

[41] Lyons PG, Micek ST, Hampton N, Kollef MH. Sepsis-associated coagulopathy severity predicts hospital mortality. Crit Care Med. 2018;46(5):736–42. 29373360 10.1097/CCM.0000000000002997

[42] Zhao H, Dong Y, Wang S, Shen J, Song Z, Xue M, Shao M. Comparison between sepsis-induced coagulopathy and sepsis-associated coagulopathy criteria in identifying sepsis-associated disseminated intravascular coagulation. World J Emerg Med. 2024;15(3):190–6. 38855376 10.5847/wjem.j.1920-8642.2024.041 PMC11153374

[43] Czempik PF, Wiórek A. Management strategies in septic coagulopathy: a review of the current literature. Healthcare. 2023;11(2):227. 36673595 10.3390/healthcare11020227 PMC9858837

[44] Nam JJ, Wong AI, Cantong D, Cook JA, Andrews Z, Levy JH. Sepsis-induced coagulopathy and disseminated intravascular coagulation: what we need to know and how to manage for prolonged casualty care. J Spec Oper Med. 2023;23(2):118–21. 37302145 10.55460/6OZC-JIOV

[45] Iba T, Helms J, Totoki T, Levy JH. Heparins may not be the optimal anticoagulants for sepsis and sepsis-associated disseminated intravascular coagulation. Semin Thromb. 2024;11. 10.1055/s-0044-1786754 10.1055/s-0044-1786754 38733977

[46] Iba T, Umemura Y, Wada H, Levy JH. Roles of coagulation abnormalities and microthrombosis in sepsis: pathophysiology, diagnosis, and treatment. Arch Med Res. 2021;52(8):788–97. 34344558 10.1016/j.arcmed.2021.07.003

[47] Yamakawa K, Levy JH, Iba T. Recombinant human soluble thrombomodulin in patients with sepsis-associated coagulopathy (SCARLET): an updated meta-analysis. Crit Care. 2019;23(1):302. 31488189 10.1186/s13054-019-2587-2 PMC6729086

[48] Tsuchida T, Makino Y, Wada T, Ushio N, Totoki T, Fujie N, Yasuo S, Matsuoka T, Koami H, Yamakawa K, Iba T. Efficacy of antithrombin administration for patients with sepsis: a systematic review, meta-analysis, and meta-regression. Acute Med Surg. 2024;11(1):e950. 38638892 10.1002/ams2.950 PMC11024450

[49] Egi M, Ogura H, Yatabe T, Atagi K, Inoue S, Iba T, Kakihana Y, Kawasaki T, Kushimoto S, Kuroda Y, Kotani J, Shime N, Taniguchi T, Tsuruta R, Doi K, Doi M, Nakada TA, Nakane M, Fujishima S, Hosokawa N, Masuda Y, Matsushima A, Matsuda N, Yamakawa K, Hara Y, Sakuraya M, Ohshimo S, Aoki Y, Inada M, Umemura Y, Kawai Y, Kondo Y, Saito H, Taito S, Takeda C, Terayama T, Tohira H, Hashimoto H, Hayashida K, Hifumi T, Hirose T, Fukuda T, Fujii T, Miura S, Yasuda H, Abe T, Andoh K, Iida Y, Ishihara T, Ide K, Ito K, Ito Y, Inata Y, Utsunomiya A, Unoki T, Endo K, Ouchi A, Ozaki M, Ono S, Katsura M, Kawaguchi A, Kawamura Y, Kudo D, Kubo K, Kurahashi K, Sakuramoto H, Shimoyama A, Suzuki T, Sekine S, Sekino M, Takahashi N, Takahashi S, Takahashi H, Tagami T, Tajima G, Tatsumi H, Tani M, Tsuchiya A, Tsutsumi Y, Naito T, Nagae M, Nagasawa I, Nakamura K, Nishimura T, Nunomiya S, Norisue Y, Hashimoto S, Hasegawa D, Hatakeyama J, Hara N, Higashibeppu N, Furushima N, Furusono H, Matsuishi Y, Matsuyama T, Minematsu Y, Miyashita R, Miyatake Y, Moriyasu M, Yamada T, Yamada H, Yamamoto R, Yoshida T, Yoshida Y, Yoshimura J, Yotsumoto R, Yonekura H, Wada T, Watanabe E, Aoki M, Asai H, Abe T, Igarashi Y, Iguchi N, Ishikawa M, Ishimaru G, Isokawa S, Itakura R, Imahase H, Imura H, Irinoda T, Uehara K, Ushio N, Umegaki T, Egawa Y, Enomoto Y, Ota K, Ohchi Y, Ohno T, Ohbe H, Oka K, Okada N, Okada Y, Okano H, Okamoto J, Okuda H, Ogura T, Onodera Y, Oyama Y, Kainuma M, Kako E, Kashiura M, Kato H, Kanaya A, Kaneko T, Kanehata K, Kano KI, Kawano H, Kikutani K, Kikuchi H, Kido T, Kimura S, Koami H, Kobashi D, Saiki I, Sakai M, Sakamoto A, Sato T, Shiga Y, Shimoto M, Shimoyama S, Shoko T, Sugawara Y, Sugita A, Suzuki S, Suzuki Y, Suhara T, Sonota K, Takauji S, Takashima K, Takahashi S, Takahashi Y, Takeshita J, Tanaka Y, Tampo A, Tsunoyama T, Tetsuhara K, Tokunaga K, Tomioka Y, Tomita K, Tominaga N, Toyosaki M, Toyoda Y, Naito H, Nagata I, Nagato T, Nakamura Y, Nakamori Y, Nahara I, Naraba H, Narita C, Nishioka N, Nishimura T, Nishiyama K, Nomura T, Haga T, Hagiwara Y, Hashimoto K, Hatachi T, Hamasaki T, Hayashi T, Hayashi M, Hayamizu A, Haraguchi G, Hirano Y, Fujii R, Fujita M, Fujimura N, Funakoshi H, Horiguchi M, Maki J, Masunaga N, Matsumura Y, Mayumi T, Minami K, Miyazaki Y, Miyamoto K, Murata T, Yanai M, Yano T, Yamada K, Yamada N, Yamamoto T, Yoshihiro S, Tanaka H, Nishida O. The Japanese clinical practice guidelines for management of sepsis and septic shock 2020 (J-SSCG 2020). Acute Med Surg. 2021;8(1):e659. 34484801 10.1002/ams2.659 PMC8390911

[50] Yamakawa K, Yoshimura J, Ito T, Hayakawa M, Hamasaki T, Fujimi S. External validation of the two newly proposed criteria for assessing coagulopathy in sepsis. Thromb Haemost. 2019;119(2):203–12. 30593085 10.1055/s-0038-1676610

[51] Tullo G, Covino M, Carbone L, Dico FL, Corsini G, Piccioni A, Polla DD, Petrucci M, Sandroni C, Simeoni B, Gasbarrini A, Franceschi F. Sepsis-induced coagulopathy (SIC) score is an independent predictor of mortality and overt-disseminated intravascular coagulation in emergency department patients with sepsis. Signa Vitae. 2024;20(6):33–43.

[52] Helms J, Severac F, Merdji H, Clere-Jehl R, François B, Mercier E, Quenot JP, Meziani F, CRICS TRIGGERSEP Group (Clinical Research in Intensive Care and Sepsis Trial Group for Global. Evaluation and research in sepsis). Performances of disseminated intravascular coagulation scoring systems in septic shock patients. Ann Intensive Care. 2020;10(1):92. 32651674 10.1186/s13613-020-00704-5 PMC7352012

[53] Ding R, Wang Z, Lin Y, Liu B, Zhang Z, Ma X. Comparison of a new criteria for sepsis-induced coagulopathy and international society on thrombosis and haemostasis disseminated intravascular coagulation score in critically ill patients with sepsis 3.0: a retrospective study. Blood Coagul Fibrinolysis. 2018;29(6):551–8. 30015646 10.1097/MBC.0000000000000755 PMC6133197

[54] Ortega-Martorell S, Olier I, Johnston BW, Welters ID. Sepsis-induced coagulopathy is associated with new episodes of atrial fibrillation in patients admitted to critical care in sinus rhythm. Front Med. 2023;10:1230854. 10.3389/fmed.2023.1230854 PMC10540306 37780563

[55] Williams B, Zou L, Pittet JF, Chao W. Sepsis-induced coagulopathy: a comprehensive narrative review of pathophysiology, clinical presentation, diagnosis, and management strategies. Anesth Analg. 2024;138(4):696–711. 38324297 10.1213/ANE.0000000000006888 PMC10916756

[56] Prado Y, Tapia P, Eltit F, Reyes-Martínez C, Feijóo CG, Llancalahuen FM, Riedel CA, Cabello-Verrugio C, Stehberg J, Simon F. Sepsis-induced coagulopathy phenotype induced by oxidized high-density lipoprotein associated with increased mortality in septic-shock patients. Antioxidants. 2023;12(3):543. 36978791 10.3390/antiox12030543 PMC10045333

[57] Iba T, Arakawa M, Di Nisio M, Gando S, Anan H, Sato K, Ueki Y, Levy JH, Thachil J. Newly proposed sepsis-induced coagulopathy precedes international society on thrombosis and haemostasis overt-disseminated intravascular coagulation and predicts high mortality. J Intensive Care Med. 2020;35(7):643–9. 29720054 10.1177/0885066618773679

[58] Warren BL, Eid A, Singer P, Pillay SS, Carl P, Novak I, Chalupa P, Atherstone A, Pénzes I, Kübler A, Knaub S, Keinecke HO, Heinrichs H, Schindel F, Juers M, Bone RC, Opal SM. KyberSept trial study group. Caring for the critically ill patient. High-dose antithrombin III in severe sepsis: a randomized controlled trial. JAMA. 2001;286(15):1869–78. 11597289 10.1001/jama.286.15.1869

[59] Ranieri VM, Thompson BT, Barie PS, Dhainaut JF, Douglas IS, Finfer S, Gårdlund B, Marshall JC, Rhodes A, Artigas A, Payen D, Tenhunen J, Al-Khalidi HR, Thompson V, Janes J, Macias WL, Vangerow B, Williams MD, PROWESS-SHOCK Study Group. Drotrecogin alfa (activated) in adults with septic shock. N Engl J Med. 2012;366(22):2055–64. 22616830 10.1056/NEJMoa1202290

[60] Abraham E, Reinhart K, Opal S, Demeyer I, Doig C, Rodriguez AL, Beale R, Svoboda P, Laterre PF, Simon S, Light B, Spapen H, Stone J, Seibert A, Peckelsen C, De Deyne C, Postier R, Pettilä V, Sprung CL, Artigas A, Percell SR, Shu V, Zwingelstein C, Tobias J, Poole L, Stolzenbach JC, Creasey AA, OPTIMIST Trial Study Group. Efficacy and safety of tifacogin (recombinant tissue factor pathway inhibitor) in severe sepsis: a randomized controlled trial. JAMA. 2003;290(2):238–47. 12851279 10.1001/jama.290.2.238

[61] Vincent JL, Francois B, Zabolotskikh I, Daga MK, Lascarrou JB, Kirov MY, Pettilä V, Wittebole X, Meziani F, Mercier E, Lobo SM, Barie PS, Crowther M, Esmon CT, Fareed J, Gando S, Gorelick KJ, Levi M, Mira JP, Opal SM, Parrillo J, Russell JA, Saito H, Tsuruta K, Sakai T, Fineberg D. SCARLET trial group. Effect of a recombinant human soluble thrombomodulin on mortality in patients with Sepsis-Associated Coagulopathy: the SCARLET Randomized Clinical Trial. JAMA. 2019;321(20):1993–2002. 31104069 10.1001/jama.2019.5358 PMC6547077

[62] François B, Fiancette M, Helms J, Mercier E, Lascarrou JB, Kayanoki T, Tanaka K, Fineberg D, Vincent JL, Wittebole X. Efficacy and safety of human soluble thrombomodulin (ART-123) for treatment of patients in France with sepsis-associated coagulopathy: post hoc analysis of SCARLET. Ann Intensive Care. 2021;11(1):53. 33788052 10.1186/s13613-021-00842-4 PMC8012451

[63] Qi W, Liu J, Li A. Effect of anticoagulant versus non-anticoagulant therapy on mortality of sepsis-induced disseminated intravascular coagulation: a systematic review and meta-analysis. Clin Appl Thromb Hemost. 2023;29:10760296231157766. 36802946 10.1177/10760296231157766 PMC9941593

[64] Maier CL, Iba T. Designing future clinical trials for sepsis-associated disseminated intravascular coagulation. Juntendo Med J. 2024;70(2):125–8. 10.14789/jmj.JMJ24-0010-P PMC11487357 39430208

[65] Iba T, Levy JH, Warkentin TE, Thachil J, van der Poll T, Levi M, Scientific and Standardization Committee on DIC, and the Scientific and Standardization Committee on Perioperative and Critical Care of the International Society on Thrombosis and Haemostasis. Diagnosis and management of sepsis-induced coagulopathy and disseminated intravascular coagulation. J Thromb Haemost. 2019;17(11):1989–94. 31410983 10.1111/jth.14578

